# Editorial: Exercise friend or foe? for the management of oxidative stress in health and diseases Volume II

**DOI:** 10.3389/fphys.2023.1216566

**Published:** 2023-05-25

**Authors:** Anand Thirupathi, Yaodong Gu, Ricardo A. Pinho

**Affiliations:** ^1^ Faculty of Sport Science, Ningbo University, Ningbo, China; ^2^ Laboratory of Exercise Biochemistry in Health, Graduate Program in Health Sciences, School of Medicine, Pontifícia Universidade Católica do Paraná, Curitiba, Brazil

**Keywords:** oxidative stress, exercise, ROS, muscles, oxidants, and antioxidants

Physical exercise is a miracle drug for treating many diseases, including metabolic conditions, neurodegenerative diseases, and some cancers (Thornton et al., 2019). However, physical exercise’s effect in treating these diseases is linked with the production of Reactive oxygen and nitrogen species and further oxidative/nitrosative damage in the cellular environment. Indeed, from the last 4 decades, we understand how exercise softens RONS toxicity depending on an individual’s production source, types, and training status for regulating various physiological functions, such as controlling gene expression and cellular processes, rather than merely inducing oxidative damage. For example, the increase of hydrogen peroxide (H_2_O_2_) in the contracting skeletal muscles activates various redox molecules, especially thiol-based molecules in the proteins, maintaining muscle mass and improving muscle function (Jackson et al., 2020), suggesting the specific exercise that controls H_2_O_2_ within the limit may be a useful parameter for the elderly or the people who are more susceptible to muscle weakness and loss. However, exercise intensity with longer duration may further take these molecules to produce various ROS, which can overwhelm the antioxidant system and disrupts the exercise-induced adaptive benefits (Thirupathi et al., 2021a). Nevertheless, prolonged physical exercise with high intensities improves a cell’s or tissue’s tolerance rate against oxidative damage by setting a new threshold limit for the cells and tissues (Thirupathi et al., 2021b). Furthermore, this scenario can redefine the specific ROS concentration from their production site to produce the transient effect in the different cells and tissues. Also, considering the steady state of enzymatic antioxidants during different exercise types with various intensities may avoid the unnecessary use of external antioxidants and rule out the negative perception of exercise performance. Indeed, current advancements in these fields will help overcome these negative perceptions of the exercise as a primary non-invasive method for improving a healthy lifestyle rather than simply prescribing external antioxidants to overcome exercise-induced oxidative damage.

Although initial studies found that supplements such as Vitamin C and E can effectively reduce exercise-induced oxidative damage, later studies concluded that these supplements don’t reduce exercise-induced oxidative damage for increasing performance. For example, studies reported that the use of vitamins C and E hinders the increase of PGC-1 alpha and mitochondrial biogenesis in the skeletal muscles, and this can prevent exercise-induced muscle adaptation (Quintanilha and Packer, 1983; Gomez-Cabrera et al., 2005), suggesting the role of ROS as a crucial player in normal physiological functions. In addition, these supplements disturb the bearable threshold level of ROS within the specific site when used excessively. In this scenario, specific exercise protocols such as resistance type may be approached to disperse the transient effect of ROS or disperse the external administration of antioxidants from one site to another cellular site to prevent local oxidative damage and create synergy between different tissues for maintaining total redox homeostasis the entire system, mainly by activating the endogenous antioxidant system (superoxide dismutase and glutathione peroxidase) in an intensity and duration-dependent manner (post-translational modification).

The current Research Topic covers seven articles dealing with exercise effects on various physio-pathological functions, primarily related to oxidative stress as a main platform for inducing exercise benefits. An article dealt with the role of strenuous exercise as a potential candidate for inducing myocardial damage. It is reported that an increased volume of cardiac load can increase oxidative stress causing myocardial damage, suggesting the careful need for exercise before recommending it. This may be due to the increased HMGB1 and sRAGE reverted after 72 h, indicating the transient ischemic alteration rather than direct necrosis in the cardiac system (Schoenfeld et al.). Another systematic meta-analysis reported that combined exercise protocols such as aerobic and low-intensity resistance exercises dampen oxidative damage and suggested additional antioxidants are unnecessary during exercise (Ni et al.). As mentioned above, resistance exercise may disperse the local oxidative damage, and aerobic exercise may help carry this effect to the entire system. Nevertheless, this should be established with the proper oxidative damage-related mechanism. Several studies have reported the use of antioxidants for preventing exercise-induced oxidative damage. In this Research Topic, Wang et al. used taurine as an external antioxidant for preventing inflammation during running and concluded that taurine affects reducing inflammatory markers. Another study reported that treadmill exercise maintains a balanced oxidative-reductive environment within the joint by regulating inflammation (da Silva LA et al., 2023). Another study observed that physical activity improved the inflammatory status in the multiple myeloma condition (Wang et al.). Apart from these, two studies reported the effect of exercise on different biomechanical properties, which helps recommend exercise as one of the essential strategies for preventing injury.

In summary, this Research Topic addressed several aspects of physical exercise-induced oxidative damage-related diseases. In particular, this Research Topic covered how physical activity can be recommended for maintaining a healthy lifestyle. Nevertheless, revealing a specific mechanism of exercise types with different intensities can control the ROS production within the limit or induce the adaptation with the specific ROS concentration will firmly support the exercise as an essential tool for improving health benefits. Finally, we thank all the authors, reviewers, and editors who contributed and spent their precious time making this Research Topic successful.

## References

[B1] da Silva L. A. Thirupathi A. Colares M. C. Haupenthal D. P. D. S. Venturini L. M. Corrêa M. E. A. B. (2023). The effectiveness of treadmill and swimming exercise in an animal model of osteoarthritis. Front. Physiol. 14, 1101159. 10.3389/fphys.2023.1101159 36895628 PMC9990173

[B2] Gomez-Cabrera M. C. Borras C. Pallardo F. V. Sastre J. Ji L. L. Vina J. (2005). Decreasing xanthine oxidase-mediated oxidative stress prevents useful cellular adaptations to exercise in rats. J. Physiol. 567, 113–120. 10.1113/jphysiol.2004.080564 15932896 PMC1474177

[B3] Jackson M. J. Stretton C. McArdle A. (2020). Hydrogen peroxide as a signal for skeletal muscle adaptations to exercise: What do concentrations tell us about potential mechanisms? Redox Biol. 35, 101484. 10.1016/j.redox.2020.101484 32184060 PMC7284923

[B4] Quintanilha A. T. Packer L. (1983). Vitamin E, physical exercise and tissue oxidative damage. Ciba Found. Symp. 101, 56–69. 10.1002/9780470720820.ch5 6557908

[B5] Thirupathi A. Pinho R. A. Ugbolue U. C. He Y. Meng Y. Gu Y. (2021a). Effect of running exercise on oxidative stress biomarkers: A systematic review. Front. Physiol. 11, 610112. 10.3389/fphys.2020.610112 33551836 PMC7854914

[B6] Thirupathi A. Wang M. Lin J. K. Fekete G. István B. Baker J. S. (2021b). Effect of different exercise modalities on oxidative stress: A systematic review. Biomed. Res. Int. 2021, 1947928. 10.1155/2021/1947928 33628774 PMC7892233

[B7] Thornton J. Nagpal J. T. Reilly J. K. Stewart M. Petrella R. (2019). The miracle cure. BMJ 366, e001373. 10.1136/bmjsem-2022-001373 PMC9362801 35999822

